# Prevalence of dementia in selected Middle East and North Africa (MENA) countries: A systematic review and meta‐analysis

**DOI:** 10.1002/alz.71109

**Published:** 2026-01-28

**Authors:** Mohsen Sedighi, Mohammad Hasan Shahabi, Alireza Amanollahi, Khurshid Alam, Soudabeh Shemehsavar, Zahra Shirzadi, Serena Sabatini, Ahmad R. Khatoonabadi, Matthew Prina, Akram A. Hosseini, Claire V. Burley, Jennifer Dunne, Simin Mahinrad, Iman Dajani, Ralph N. Martins, Blossom C. M. Stephan, Ali Chaari, Hamid R. Sohrabi

**Affiliations:** ^1^ Trauma and Injury Research Center Iran University of Medical Sciences Tehran Iran; ^2^ Department of Psychiatry and Psychotherapy University of Marburg Marburg Germany; ^3^ Murdoch Business School, Murdoch University Murdoch WA Australia; ^4^ Centre for Healthy Ageing Health Futures Institute Murdoch University Murdoch WA Australia; ^5^ College of Science Technology Engineering and Mathematics Murdoch University Murdoch WA Australia; ^6^ Department of Neurology Massachusetts General Hospital, Harvard Medical School Boston Massachusetts USA; ^7^ Medical & Scientific Relations, Alzheimer's Association Chicago Illinois USA; ^8^ Department of Clinical Psychology and Psychobiology University of Barcelona Barcelona Spain; ^9^ School of Rehabilitation Speech Therapy Department Tehran University of Medical Sciences Tehran Iran; ^10^ Population Health Sciences Institute Newcastle University Newcastle UK; ^11^ Department of Academic Neurology Queen's Medical Centre, Nottingham University Hospitals NHS Trust Nottingham UK; ^12^ Sir Peter Mansfield Imaging Centre University of Nottingham Nottingham UK; ^13^ Dementia Centre of Excellence, enAble Institute Curtin University Perth WA Australia; ^14^ Weill Cornell Medicine Qatar Qatar Foundation Education City Doha Qatar; ^15^ School of Medical and Health Sciences Edith Cowan University Joondalup WA Australia; ^16^ Macquarie Medical School Macquarie University New South Wales Australia; ^17^ School of Psychology Murdoch University Murdoch WA Australia

**Keywords:** Alzheimer's disease, dementia, MENA, meta‐analysis, Middle East, North Africa, prevalence

## Abstract

Data on dementia epidemiology in the Middle East and North Africa (MENA) region is limited. This systematic review and meta‐analysis examined dementia prevalence across MENA. Databases were searched up to October 2024. Analyses were stratified by country and sex. Pooled prevalence was estimated using a random‐effects model with a 95% confidence interval (CI). Fifty‐two studies on the selected countries met inclusion criteria, covering 87,219 individuals with dementia from a total population of 1,045,908. The pooled prevalence was 12.16% (95% CI: 9.61–14.96) for the region and the Israel had the highest prevalence (17.00%), followed by Iran (13.20%), Turkey (11.40%), Saudi Arabia (8.34%), and Egypt (6.86%). Dementia was more common in women than men (13.84% vs. 8.69%). Dementia is prevalent in MENA, with significant variation across countries. The region's aging population highlights the need for ongoing monitoring of dementia trends.

## Highlights

- Dementia is common in the Middle East and North Africa (MENA) region, but there is a major gap in research across the region.
- The pooled prevalence of dementia in the MENA region is 12.16%.
- Dementia is more common among women (13.84%) compared to men (8.69%).
- Dementia prevalence varies significantly across MENA countries.

## 1 BACKGROUND

Alzheimer's disease and related dementias (ADRD) are the leading cause of cognitive impairment in older adults (aged ≥ 65 years) across the world.^1^ AD, vascular dementia (VaD), dementia with Lewy bodies (DLB), and frontotemporal dementia (FTD) are the most common forms of the disease and available data show that AD and VaD account for 60% and 20% of dementia cases, respectively.^2^ The estimated global number of people living with dementia was 57.6 million in 2021, with approximately 10 million new cases diagnosed each year, and the number is projected to reach 152.8 million cases by 2050.^3^ Most people (∼67%) with dementia live in low‐ and middle‐income countries (LMICs) that may not have policies in place to cope with the social and economic burdens of disease or programs of risk reduction that are necessary to impact future numbers.^4^, ^5^

Epidemiological studies have constantly shown differences in the rates of dementia among different geographical regions and ethnic populations. The higher prevalence is typically observed in rural compared to urban areas.^6^ People from the Black and Asian ethnic groups have approximately 30% higher incidence of dementia compared to individuals from the White ethnic background.^7^ According to the recent reports, the highest number of people with dementia are currently living in Asia (22.9 million), which is more than double the number reported in Europe (10.5 million) or America (9.4 million).^8^ Moreover, prevalence of dementia in Arab countries varies according to sociodemographic characteristics, aging and illiteracy are the major risk factors for dementia in these populations.^9^

**FIGURE 1:**
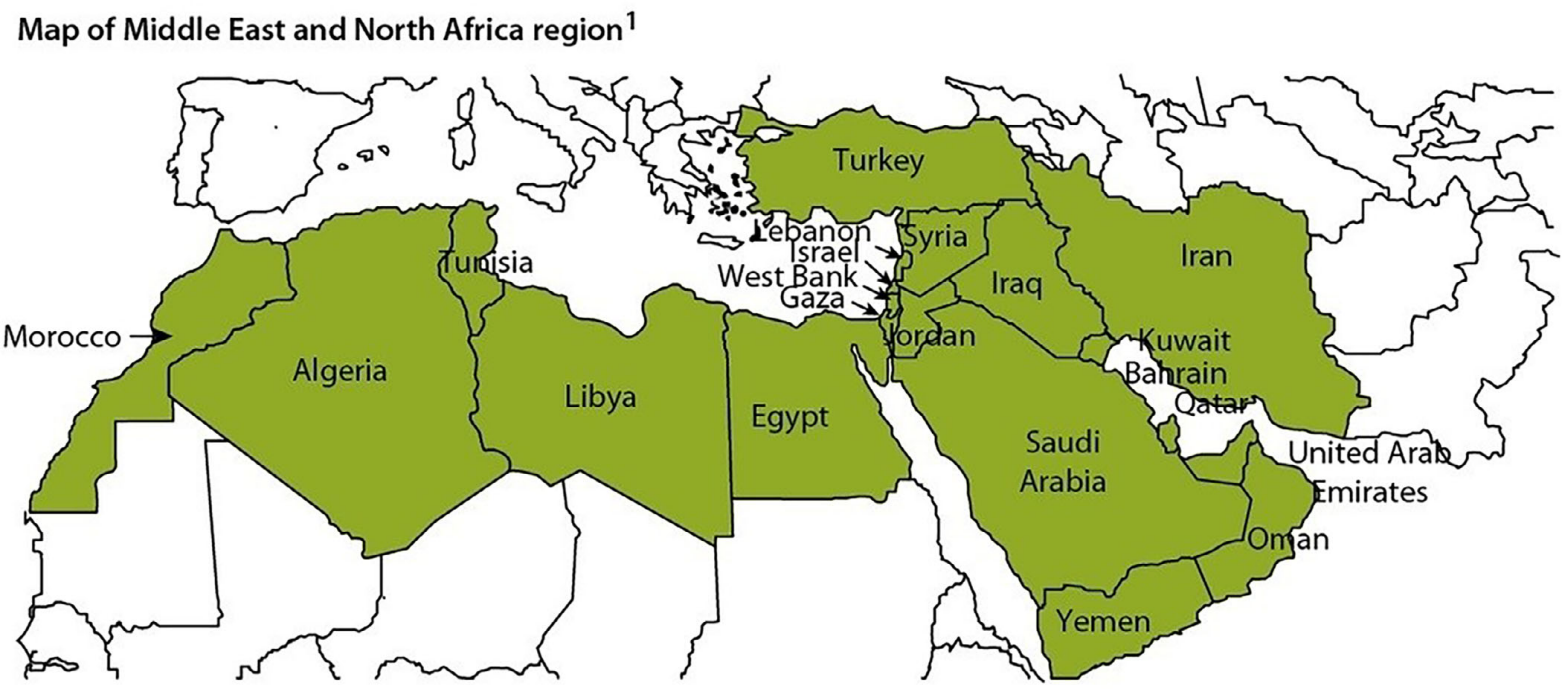
Map of the Middle East and North Africa (MENA) region.

The Middle East and North Africa (MENA) region includes countries spanning from Morocco in the West, to Iran in the East, and Turkey in the North, to Yemen in the South (Figure 1). The MENA region comprises approximately 23 countries and almost 400 million people, accounting for approximately 6% of the world's population.^10^ Previous research has reported that the age‐standardised point prevalence of AD increased by 3.0% in MENA over 1990‐2019.^11^ Currently, dementia does not seem to be an epidemic, nor is its risk looming over MENA countries, except for Israel, where high life expectancy and certain Arab communities in the country may contribute to higher risk.^12^ Nevertheless, global prevalence of dementia has doubled every 20 years globally and is projected to increase by 438% in the MENA region during 2010‐2050.^13^

Within the last decades, MENA countries have experienced substantial socioeconomic changes, including improvements in healthcare systems, further access to education, and lifestyle changes specifically in diet, air pollution, and physical activity that are also risk factors for dementia.^14^ However, unique combination of rapid urbanization, diverse socioeconomic landscapes, and political instability in the MENA region complicates efforts to address dementia.^15^ Despite the implications for health policy makers and health providers, limited amount within the last few decades, MENA countries have experienced substantial socioeconomic changes, including improvements in healthcare systems, greater access to education, and lifestyle changes—specifically in diet, air pollution, and physical activity—which are also risk factors for dementia.^14^ However, the unique combination of rapid urbanization, diverse socioeconomic landscapes, and political instability in the MENA region complicates efforts to address dementia.^15^ Despite the implications for health policymakers and providers, limited data have reported the burden and prevalence of dementia in the MENA region. In addition, prior reviews of community‐based studies on the epidemiology of dementia in the MENA region have mainly focused on Arab countries, while Turkey, Israel, and Iran have not been included. Therefore, to address this gap in the literature, we conducted a systematic review and meta‐analysis of published studies examining the prevalence of dementia across most countries in the MENA region, including publications from non‐Arab countries.

## 2 METHODS

### 2.1 Study design

This study was a systematic review and meta‐analysis according to the Preferred Reporting Items in Systematic Reviews and Meta‐Analyses (PRISMA) guidelines,^16^ and the protocol of the study was registered in PROSPERO (CRD42023478070).

### 2.2 Search strategy

A comprehensive search was performed to retrieve the most relevant published literature. We selected databases of Scopus, Web of Science, PubMed, Cochrane, and Google Scholar search engine. Moreover, to extend the scope of the literature search, a manual review of the reference lists of selected publications and cited publications was conducted. The final search was updated on October 30, 2024, before data analysis. A combination of Medical Subject Heading (MeSH) terms and search terms was utilized, including “AND” and “OR” operators to retrieve the targeted results. The key terms used in the search include “Prevalence”; “Incidence”; “Dementia”; “Alzheimer's Disease (AD)”; “MENA region” and the name of all 23 countries in the MENA region (Supplementary data). Only two restrictions were applied during the search and selection of studies, including the selection of human studies and English‐language publications.

### 2.3 Eligibility criteria

The literature search was restricted to articles published in English that met the following inclusion criteria: (1) original research; (2) population‐based; (3) observational design (prospective, retrospective, cross‐sectional); (4) reported estimate of dementia. Exclusion criteria were case reports and case series, clinical trials, review or editorial articles, non‐English language publications, and non‐relevant regions and populations.

### 2.4 Study selection, data items, and data collection

After removing duplicate records in the screening phase, two independent researchers (M.H.S and A.A) separately reviewed title and abstract of the remaining studies, and each of them extracted all data from articles. The outcome of the screening step demonstrated no disagreements or inconsistencies within the selection of studies. Relevant information was extracted using a customized datasheet containing the author's name, year of publication, participants’ mean age, gender, country, study design, sample sizes, and key study findings. Any disagreement at each step was checked by a third researcher (M.S).

### 2.5 Synthesis of results

The prevalence estimates and its 95% confidence intervals (95% CI) were calculated based on the number of dementia cases along with the total sample size reported in each study. The pooled estimate of prevalence of dementia was calculated using a random effects model based on the DerSimonian‐Laird method.^17^ Also, we performed subgroup analysis based on publication year, country, and gender using the random effects model and countries with three or more studies were considered for stratification analysis. Sensitivity analysis was performed to assess the influence of each study on the pooled estimates, and heterogeneity was assessed using the I^2^ and chi‐squared tests. To evaluate publication bias, Egger's test, Doi plot and Luis Furuya‐Kanamori (LFK) index were performed to detect and quantify symmetry of study effects on Doi plot. The LFK value within ± 1, between ± 1 and ± 2, and greater than ± 2 can be interpreted respectively as no asymmetry, minor asymmetry, and major asymmetry in Doi plot.^18^ Country and gender stratifications were applied to reduce heterogeneity. Age and publication year were also used to control for heterogeneity in meta‐regression. All statistical analyses for meta‐analysis were conducted using Stata (version 17.0; Stata Corporation).

## 3 RESULTS

**FIGURE 2:**
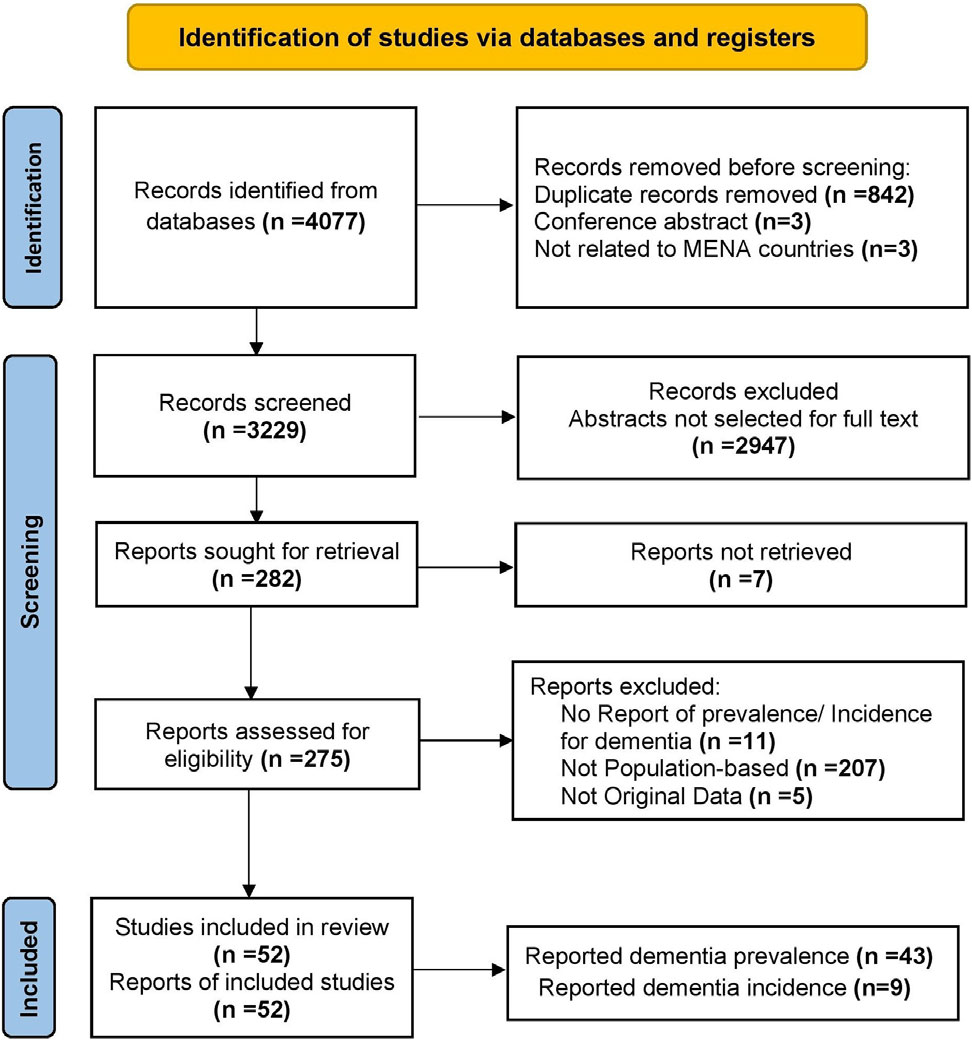
PRISMA flow diagram of study selection. PRISMA, Preferred Reporting Items in Systematic Reviews and Meta‐Analyses

The literature search resulted in 4,077 publications, including duplicates. After first screening, 848 publications were excluded as clearly ineligible, leaving 3,229 for further review. In the second step, title and abstract of publications were screened and 2,947 articles were not eligible for full‐text review. Of the remaining 282 articles, 7 did not have the full text and 223 were excluded because of such reasons as not reporting estimate of dementia (11), being hospital or clinic‐based studies (207), and lack of no original data (5). Finally, a total of 52 population‐based studies^19^, ^20^, ^21^, ^22^, ^23^, ^24^, ^25^, ^26^, ^27^, ^28^, ^29^, ^30^, ^31^, ^32^, ^33^, ^34^, ^35^, ^36^, ^37^, ^38^, ^39^, ^40^, ^41^, ^42^, ^43^, ^44^, ^45^, ^46^, ^47^, ^48^, ^49^, ^50^, ^51^, ^52^, ^53^, ^54^, ^55^, ^56^, ^57^, ^58^, ^59^, ^60^, ^61^, ^62^, ^63^, ^64^, ^65^, ^66^, ^67^, ^68^, ^69^, ^70^ met the eligibility criteria for inclusion in the meta‐analysis (Figure 2) and their characteristics are summarized in Table 1.

**TABLE 1:** Demographic characteristics of studies included in this systematic review and meta‐analysis.

ID	Study	Year	Country	Design	Screening tool	Sample size	Age	Gender	No. of dementia cases	Participants	Risk factor	Comorbidities
1	Belbari^19^	2021	Algeria	Cross‐sectional	DSM‐4	3896	–	1406 (male) 2490 (female)	75 (male) 117 (female)	Older adults aged ≥ 60 years	Smoking, drinking Family history, Physical inactivity	–
2	Farrag^20^	1998	Egypt	Cross‐sectional	MMSE	2000	–	1027 (male) 973 (female)	47 (male) 43 (female)	Older adults aged ≥ 60 years	–	–
3	El‐Tallawy^21^	2010	Egypt	Prospective	–	62583	–	–	8173	All inhabitants	–	–
4	El‐Tallawy^22^	2012	Egypt	Cross‐sectional	MMSE	8173	–	4456 (male) 3717 (female)	164	Older adults aged ≥ 50 years	Smoking Family history	HTN, DM
5	El‐Tallawy^23^	2013	Egypt	Cross‐sectional	–	33285	–	16428 (male) 16857 (female)	7626	All inhabitants	–	–
6	Khedr^24^	2015	Egypt	Cross‐sectional	MMSE	691	–	340 (male) 351 (female)	47	Older adults aged ≥ 50 years	Illiteracy	DM, HTN, IHD, CVA, HLP
7	El‐Tallawy^25^	2019	Egypt	Cross‐sectional	MMSE DSM‐5	12508	–	6888 (male) 5620 (female)	55 (male) 71 (female)	Older adults aged ≥ 50 years	Smoking Family history	HTN, DM, HLP
8	Mohammadi^26^	2012	Iran	Cross‐sectional	CDR	804		428 (male) 376 (female)	84 (male) 71 (female)	Older adults aged ≥ 65 years	Illiteracy, Smoking	HTN, DM
9	Ghaderpanahi^27^	2012	Iran	Cross‐sectional	MMSE	108	84.6 ± 4.3	34 (male) 74 (female)	71	Older adults aged ≥ 80 years	Obesity	–
10	Hosseini^28^	2014	Iran	Prospective	–	1616	69.37 ± 7.42	883 (male) 733 (female)	29	Older adults aged ≥ 60 years	–	–
11	Sharifi^29^	2016	Iran	Cross‐sectional	–	1257	69.2 ± 3	592 (male) 665 (female)	41 (male) 58 (female)	Older adults aged ≥ 60 years	Illiteracy, Smoking	HTN, DM
12	Ebrahimi^30^	2021	Iran	Cross‐sectional	MMSE	4191	–	1991 (male) 2200 (female)	8 (male) 18 (female)	Older adults aged ≥ 50 years	Smoking Family history	DM, HTN, HLP
13	Ritchie^31^	1989	Israel	Cross‐sectional		78	74 ± 9.5	–	9	Older adults aged ≥ 65 years	–	–
14	Kahana^32^	2003	Israel	Cross‐sectional	DSM III‐R	1501	80.9 ± 7	668 (male) 883 (female)	55 (male) 110 (female)	Older adults aged ≥ 75 years	Illiteracy, Smoking Family history	DM, HTN, HLP
15	Barak^33^	2003	Israel	Cross‐sectional	MMSE	98	71.79 ± 5.3	94 (male) 4 (female)	15	Elderly homeless people	Drinking	IHD, UTI, COPD
16	Schnaider Beeri^34^	2004	Israel	Prospective	MMSE	1892	44.5 ± 12	1892 male	309	Men aged 40 to 65 years		DM
17	Bowirrat^35^	2006	Israel	Cross‐sectional	DSM‐5	823	–	363 (male) 461 (female)	83 (male) 134 (female)	Older adults aged ≥ 60 years	Smoking	HTN, DM Depression
18	Wertman^36^	2007	Israel	Retrospective	MMSE	998	76 ± 6	459 (male) 539 (female)	192	Older adults aged ≥ 65 years	Illiteracy	–
19	Beeri^37^	2008	Israel	Prospective	MMSE	1628	44.5 ± 4.1	1628 (Men)	308	Men aged 40 to 65 years	–	–
20	Israeli‐Korn^38^	2010	Israel	Cross‐sectional	MMSE	756	73 ± 6	379 (male) 375 (female)	111 (male) 199 (female)	Older adults aged ≥ 65 years	–	DM, HTN, CVD
21	Afgin^39^	2012	Israel	Cross‐sectional	MMSE	944	–	466 (male) 478 (female)	447	Older adults aged ≥ 65 years	Illiteracy	–
22	Ravona Springer^40^	2013	Israel	Cross‐sectional	DSM‐5	1620	–	1620 (men)	295	Healthy working men	Smoking Obesity	–
23	Inzelberg^41^	2015	Israel	Cross‐sectional	–	857	73 ± 6	451 (male) 406 (female)	392	Older adults aged ≥ 65 years	–	DM, HTN, HLP
24	Garfinkel^42^	2018	Israel	Prospective	MMSE	177	82.89 ± 5.7	64 (male) 113 (female)	38	Older adults aged ≥ 65 years	–	DM, HTN, CVD
25	Kodesh^43^	2019	Israel	Retrospective	–	122829	–	–	8153	Older adults aged ≥ 60 years	–	DM, IHD, Mental illnesses
26	Fund^44^	2019	Israel	Cross‐sectional	–	73528	78.3 ± 4.6	32286 (male) 41242 (female)	9744	Holocaust survivors	Obesity	DM, HTN, CVD
27	Kodesh^45^	2019	Israel	Prospective	–	71515	71.4 ± 7.4	35631 (male) 35884 (female)	2176	Older adults aged ≥ 60 years	–	COPD, DM, CAD
28	Kodesh^46^	2019	Israel	Retrospective	–	51752	60.4 ± 8.2	23548 (male) 28204 (female)	5584	Israeli residents	Obesity, Sleep disorder	DM, PTSD
29	Kodesh^47^	2020	Israel	Prospective	–	94120	69.8 ± 7.1	43747 (male) 50373 (female)	6026	Older adults aged 60 to 90 years	Smoking Obesity	DM, HTN, CVD
30	Ben Zaken^48^	2020	Israel	Retrospective	–	44532	36.13 ± 23.8	–	1101	All inhabitants	–	–
31	Green^49^	2021	Israel	Cross‐sectional	–	22563	39.2 ± 22.5	11635 (male) 10928 (female)	1066	Members of a health system organization	Smoking Obesity	HTN, DM, IHD Depression
32	Abu Fanne^50^	2022	Israel	Retrospective	–	73254	50.6 ± 18.9	26903 (male) 46351 (female)	3203	Members of a health system organization		HTN, DM Depression
33	Weiss^51^	2022	Israel	Retrospective	MMSE MoCA	48632	83.2 ± 6.4	20225 (male) 28407 (female)	8848	Older adults aged ≥ 65 years	–	–
34	Weiss^52^	2022	Israel	Prospective	MMSE MoCA	36951	83 ± 6.3	14948 (male) 22003 (female)	2554 (male) 4626 (female)	Older adults aged ≥ 65 years	Smoking Obesity	HTN, DM, CVD
35	Levine^53^	2023	Israel	Prospective	–	109218	57.7 ± 5.5	52744 (male) 56474 (female)	7726	Older adults aged 51 to 70 years	–	ADHD
36	Levine^54^	2023	Israel	Prospective	–	91307	68.29 ± 6.38	42713 (male) 48594 (female)	5298	Older adults aged ≥ 60 years	Opioid use	–
37	Phung^55^	2017	Lebanon	Cross‐sectional		502	72.5 ± 7.2	220 (male) 282 (female)	6 (male) 31 (female)	Older adults aged ≥ 65 years	Illiteracy	–
38	Al Rajeh^56^	1993	Saudi Arabia	Cross‐sectional	–	23217	–	11554 (male) 11663 (female)	10	Total population	–	–
39	Althaiban^57^	2023	Saudi Arabia	Cross‐sectional	–	271	68.8 ± 8.7	138 (male) 133 (female)	19	Older adults aged ≥ 60 years	–	–
40	Alqurashi^58^	2024	Saudi Arabia	Cross‐sectional	SLUMS	343	65 ± 9	233 (male) 110 (female)	61 (male) 47 (female)	Older adults aged ≥ 60 years	Illiteracy	–
41	Al‐Sabahi^59^	2014	Oman	Retrospective	–	1561		806 (male) 860 (female)	395	Older adults aged ≥ 60 years	Physical inactivity	Depression
42	Keskinoglu^60^	2006	Turkey	Cross‐sectional	MMSE	201	70.8 ± 6.45	78 (male) 123 (female)	8 (male) 38 (female)	Older adults aged ≥ 65 years	–	–
43	Gurvit^61^	2008	Turkey	Cross‐sectional	–	1019	74.9 ± 5	395 (male) 624 (female)	29 (male) 64 (female)	Older adults aged ≥ 70 years	Illiteracy	–
44	Arslantaş^62^	2009	Turkey	Cross‐sectional	MMSE CDR	3100	–	1277 (male) 1823 (female)	68 (male) 194 (female)	Older adults aged ≥ 55 years	Family history, illiteracy	–
45	Keskinoǧlu^63^	2013	Turkey	Cross‐sectional	MMSE	490	71.8 ± 6.5	200 (male) 290 (female)	14 (male) 49 (female)	Older adults aged ≥ 65 years	Illiteracy	CVD
46	Ertekin^64^	2015	Turkey	Cross‐sectional	–	445	71.7 ± 6.8	230 (male) 225 (female)	8 (male) 21 (female)	Older adults aged ≥ 65 years	Family history, illiteracy	DM, CVD
47	Dinç Horasan^65^	2019	Turkey	Cross‐sectional	–	18477	68.8 ± 28.1	8748 (male) 9729 (female)	129	People aged ≥ 15 years	–	DM, HTN, CVD
48	Yalcintas^66^	2021	Turkey	Prospective	MMSE	399	73.59 ± 6.44	399	224	Women aged ≥ 65 years	–	DM, HTN, HLP
49	Phiri^67^	2022	Turkey	Cross‐sectional	–	11993	–	5498 (male) 6495 (female)	116	People aged ≥ 15 years	–	Depression
50	Sütlü^68^	2022	Turkey	Cross‐sectional	–	383	–	104 (male) 279 (female)	36	Older adults aged ≥ 65 years	Illiteracy, Smoking Drinking	DM, CVD, COPD
51	Bozkurt^69^	2023	Turkey	Cross‐sectional	Mini‐Cog	204	75.5 ± 7.3	94 (male) 110 (female)	17 (male) 15 (female)	Older adults aged ≥ 60 years	–	–
52	Ghubash^70^	2004	UAE	Cross‐sectional	–	610	68.6 ± 8.3	347 (male) 263 (female)	22	Older adults aged ≥ 60 years		

**Abbreviation**: ADHD, attention‐deficit/hyperactivity disorder; CDR, Clinical Dementia Rating; COPD, chronic obstructive pulmonary disease; CVD, cardiovascular disease; DM, Diabetes mellitus; DSM, Diagnostic and Statistical Manual of Mental Disorders; HLP, hyperlipidemia; HTN, hypertension; IHD, ischemic heart disease; MMSE, Mini‐Mental State Examination; MoCA, Montreal Cognitive Assessment; PTSD, post‐traumatic stress disorder; SLUMS, Saint Louis University Mental Status; UAE, United Arab Emirates.

### 3.1 Study characteristics

Of the 52 included studies, 10 were from Turkey, 24 from Israel, 5 from Iran, 6 from Egypt, 3 from Saudi Arabia, and 4 from Algeria, Lebanon, Oman, and 1 study from the United Arab Emirates (UAE). Among the included studies, 43 reported prevalence that were considered for pooled estimation of prevalence and 9 reported incidences of dementia that were excluded from final analysis Data from other MENA countries, including Qatar, Jordan, Tunisia, Iraq, Kuwait, Libya, and Morocco were only available on hospital‐based studies and therefore were discarded. The Mini‐Mental State Examination (MMSE) and Diagnostic and Statistical Manual of Mental Disorders (DSM; DSM‐III, DSM‐IV, or DSM‐V) were used in most studies to identify patients with dementia.

### 3.2 Pooled prevalence of dementia

We included 1,045,908 participants from 52 studies, of whom 87,219 had dementia. Of them, 786 (1%) cases were classified as mild cognitive impairment (MCI), 85,016 (97%) with different types of dementia such as VaD, DLB, and FTD, and 1,417 (2%) with AD. Among the included studies, 43 studies reported dementia prevalence, therefore, pooled prevalence of dementia based on 43 studies was 12.16% (95% CI: 9.61‐14.96) in the MENA region. (Table 2).

**TABLE 2:** Subgroup analysis of the dementia prevalence and incidence based on countries.

Measure	MENA country	No. of studies	Pooled estimate (95% CI)	Heterogeneity		Publication bias
				I^2^ (%)	Chi‐squared	Egger's
Prevalence	Total	43	12.16 (9.61‐ 14.96)	99.89	<0.001	<0.001
	Algeria	1	4.93 (4.27‐5.66)	–	–	–
	Egypt	6	6.86 (1.85‐ 14.69)	99.94	<0.001	0.301
	Iran	5	13.20 (4.12‐ 26.29)	99.45	<0.001	<0.001
	Israel	15	17 (13.01‐ 21.40)	99.91	<0.001	<0.001
	Saudi Arabia	3	8.34 (0.02‐ 37.67)	94.12	<0.001	0.451
	Lebanon	1	7.37 (5.24‐ 10.02)	–	–	–
	Oman	1	25.3 (23.16‐ 27.54)	–	–	–
	Turkey	10	11.40 (6.37‐ 17.63)	99.52	<0.001	<0.001
	UAE	1	3.61 (2.27‐ 5.41)	–	–	–
Incidence	Israel	9	11.05 (7.99 – 14.53)	99.91	<0.001	0.287

Abbreviations: CI, confidence interval; MENA, Middle East and North Africa.

### 3.3 Subgroup analysis

**FIGURE 3:**
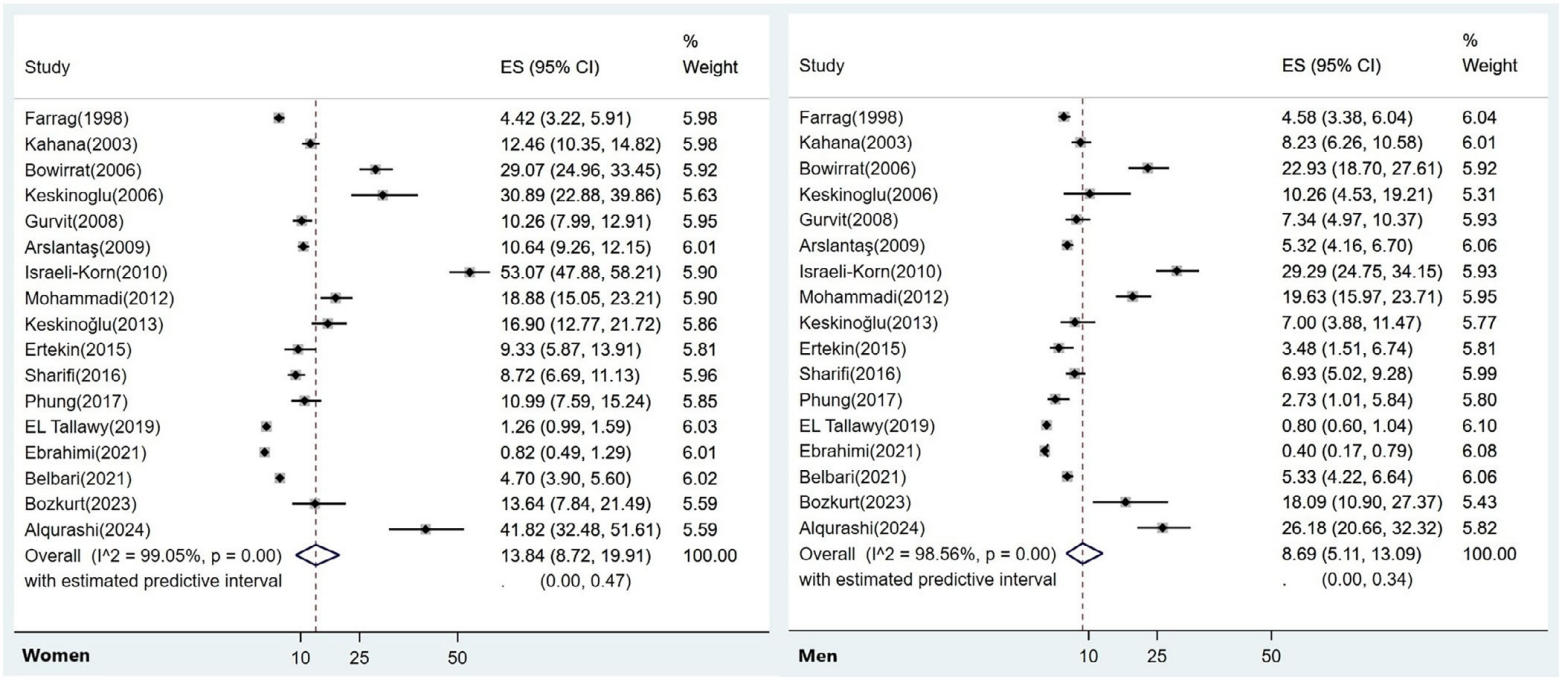
Prevalence of dementia in the MENA region stratified by gender. There was a higher prevalence of dementia among women than men (13.84% vs. 8.69%). MENA, Middle East and North Africa.

As explained in the methodology section, MENA countries with an appropriate number of publications were considered for subgroup analysis. Results of subgroup analysis based on publication year revealed pooled prevalence of 7.2% for 1998‐2005, 19.1% for 2006‐2015, and 11.5% for 2016‐2024. In terms of country subgroup, pooled prevalence of dementia in Israel was 17.00% (95% CI: 13.01‐21.40), which was the highest. For Iran, the pooled prevalence was 13.20% (95% CI: 4.12‐26.29), and the estimate for Turkey was 11.40% (95% CI: 6.37–17.63), followed by 8.34% (95% CI: 0.02‐37.67) for Saudi Arabia and 6.86% (95% CI: 1.85–14.69) for Egypt (Table 2). According to subgroup analysis for sex based on 17 studies, the pooled estimate of dementia in men was 8.69% (95% CI: 5.11‐ 13.09) and the estimated value for women was 13.84% (95% CI: 8.72‐ 19.91), as represented in Figure 3.

### 3.4 Heterogeneity and meta‐regression

Heterogeneity was observed across studies, both in the pooled estimates and within subgroups such as country and gender, as presented in Tables 2 and 3. Additionally, age did not significantly reduce I^2^ (97.85%), even when considering the mean age of participants across the studies. The results of multiple meta‐regression showed a coefficient (β) of 0.005 (95% CI: 0.003–0.006; *p* < 0.001) for age and 0.003 (95% CI: 0.0003–0.007; *p* = 0.078) for publication year.

**TABLE 3:** Subgroup analysis of dementia prevalence based on sex in MENA countries

Sex	No. of studies	Pooled effect size (95% CI)	Heterogeneity		Publication bias
			I^2^ (%)	Chi‐squared	Egger's
Male	17	8.69 (5.11‐ 13.09)	99.43	0.001	0.711
Female	17	13.84 (8.72‐ 19.91)	99.57	0.001	0.679

Abbreviations: MENA, Middle East and North Africa.

### 3.5 Publication bias and sensitivity analysis

**FIGURE 4:**
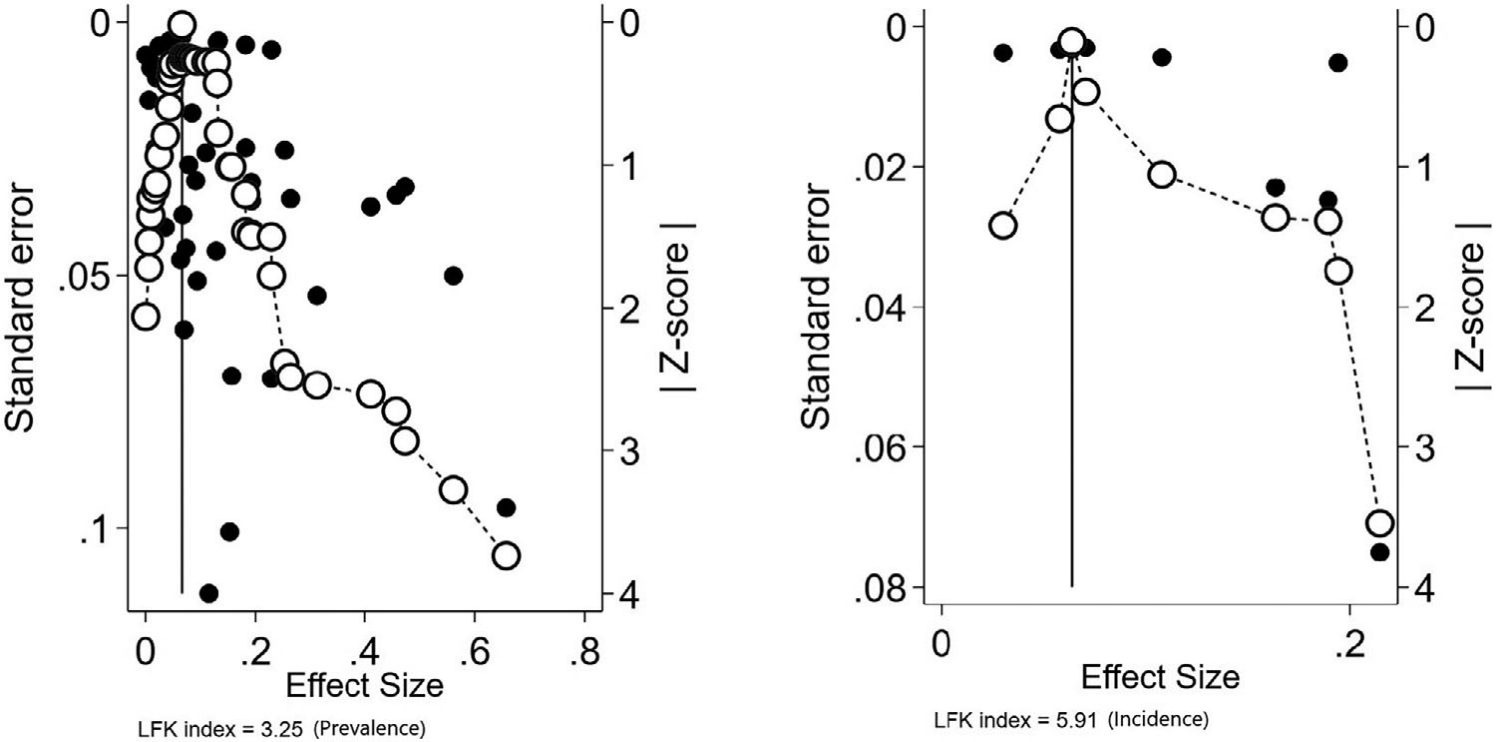
Doi plot and LFK index for assessing publication bias for prevalence of dementia in the MENA region. LFK, Luis Furuya‐Kanamori; MENA, Middle East and North Africa.

**FIGURE 5:**
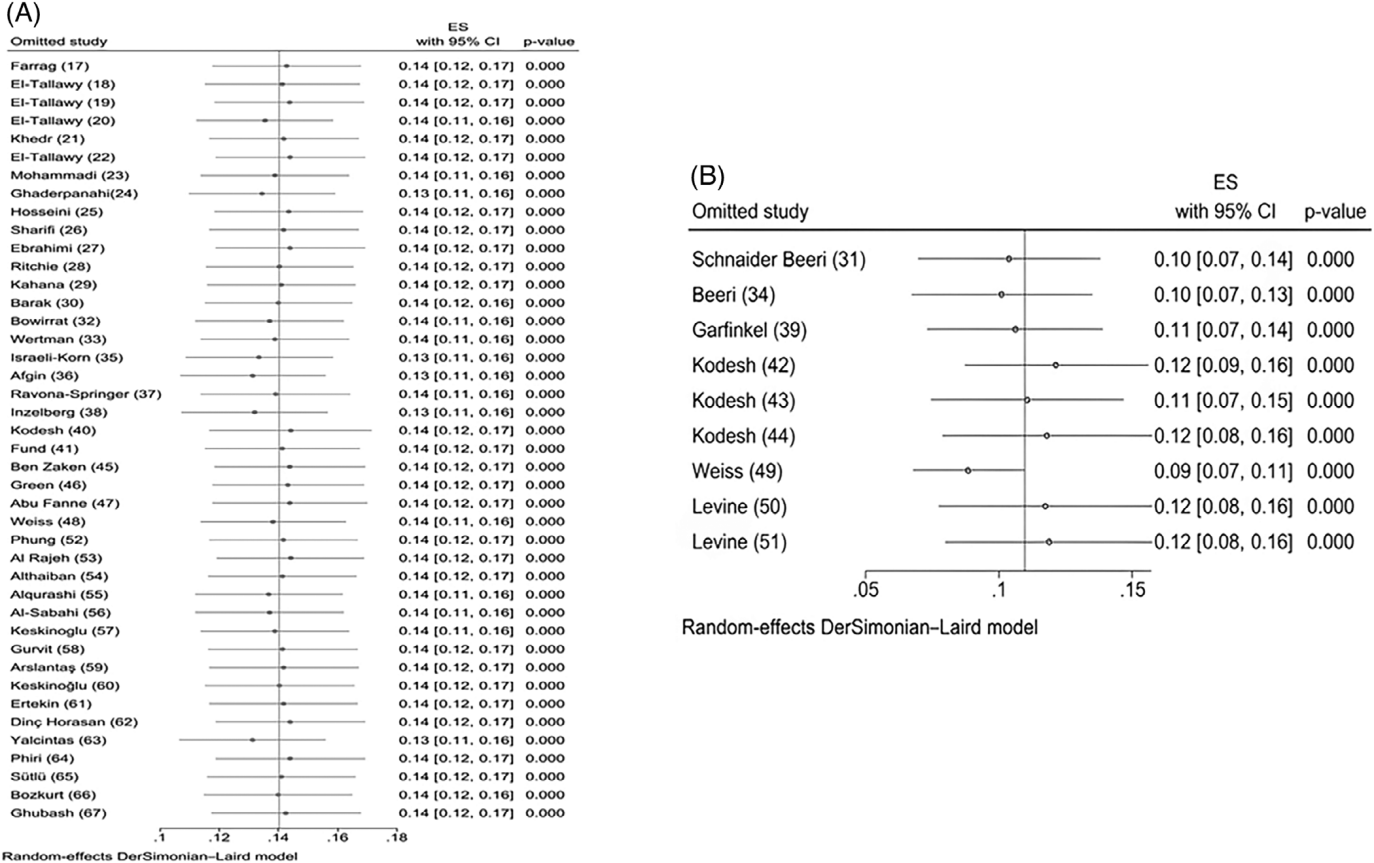
Sensitivity analysis of the studies included in the systematic review and meta‐analysis on the prevalence of dementia.

Publication bias was assessed for prevalence and Doi plot revealed a major asymmetry (LFK index = 3.25), indicating potential publication bias in favour of studies reporting a higher prevalence (Figure 4). This variation is attributed to the review design, which included prevalence studies, where the results of all studies were above one, resulting in an asymmetrical plot. Sensitivity analysis was performed to assess the robustness of the meta‐analyses by examining the influence of each study on the pooled estimates of dementia. Our results revealed that no individual study altered the overall pooled estimate for prevalence (Figure 5).

## 4 DISCUSSION

The percentage of older people in the MENA region is expected to increase and rate of dementia in this region is rising due to a combination of factors, including aging population, changing lifestyle factors, and improved life expectancy with significant societal and financial implications for all constituent countries.^71^ The 2021 Global Burden of Disease (GBD) report has shown the age‐standardized prevalence rate decreased by 4.9% in comparison to 1990 in the MENA region. Likewise, the age‐standardized death and DALY rates were 8.6% and 7.7% lower than in 1990, respectively.^72^

The current meta‐analysis study provides estimates of dementia prevalence in the MENA region with reasonable precision and offers overall estimates as well as subgroup analyses by country and sex. This study demonstrates that dementia estimates vary by country and sex, suggesting inter‐country variations even in the same region. Therefore, while global and regional GBD estimates as well as prevalence rates of dementia are informative and valuable, minimizing the risk and preventing dementia at any level of prevention requires detailed, country‐specific action plans informed by distinct lifestyle, modifiable risk factors, and cultural/societal background. Future research can inform whether such country or region‐specific approach is feasible or general and global approach are similar impactful and practical.

It has been reported that regional estimates of dementia prevalence in people aged 60 years and over range from 5.5% to 11.3% across the world.^73^ Results from a prior study on dementia prevalence in Arab countries reported rates ranging from 1.1% to 2.3% among individuals aged 50 and older, and from 13.5% to 18.5% among those aged 80 years old and above.^9^ In our meta‐analysis, the pooled estimate of dementia prevalence in MENA countries was 12.16%, which is higher than the estimates of dementia in other geographical regions worldwide.^74^ However, sub group analysis revealed a fluctuation in estimated dementia and we observed a decrease of 7.6% in prevalence between 2006–2015 and 2016–2024 that could be due to the difference in age of included participants and sampling strategy in the studies. The estimated prevalence of dementia for those aged over 60 years ranges from 3.2% to 4.8% in Asian regions, 6.2% in Europe, 6.4% in Australasia, and 6.5% in American countries.^75^ This difference may be attributed to the age and geographic region. However, as noted below, our reported findings are in line with dementia projections for LMIC for the next 25 to 30 years.^76^ On the other hand, prevalence of dementia risk factors such as obesity, raised blood glucose, hypertension, and smoking in MENA countries is much higher than the worldwide average.^77^ There has been an increasing rate of obesity and tobacco smoking, especially among women and adolescents, which are potential risk factors of dementia and significantly contribute to the accelerated rise in dementia prevalence.^78^ Cardiovascular disorders, diabetes mellitus, and mental disorders account for more than 60% of the non‐communicable disease burden, especially in high‐income countries in the MENA region.^77^, ^78^ However, as seen in other meta‐analyses,^79^ confounders arising from differences in survival length, the distribution of risk/protective factors and design/methodology (e.g., sampling and dementia diagnostic criteria) within the included studies were inevitable in our findings.

The GBD 2021 studies have reported that the number of dementia cases in MENA was 2.68 million cases (98% UI: 2.32‐3.04 million) and the age‐standardized point prevalence of dementia was 772.7 per 100,000 population in the region (95% UI 671.2‐877.6).^80^, ^81^ The age‐standardized death rate of dementia was 25.6 per 100,000 population and DALY rate was 476.3 per 100,000 population, with the highest value in Afghanistan (577.72) and the lowest value in Jordan (450.02).^81^ According to GBD 2019, the number of dementia cases in MENA is projected to increase to 13.83 million (367%) by 2050 and this figure will be the largest increase compared to any other region.^81^ Among the MENA countries, the highest projected burden will be in Qatar (1926%), followed by the UAE (1795%) and Bahrain (1084%). This higher burden in the MENA region is attributed to changes in the growth of population followed by increased life expectancy, aging population, dementia‐related risk factors, and education.^81^

Our study has demonstrated that dementia estimate was higher in women than in men (13.84% vs 8.69%) and supports prior findings reported both regionally^3^, ^82^ and globally.^76^, ^83^ Nevertheless, several cohorts from Europe,^84^ North America,^85^, ^86^ and Latin America^87^ have reported a similar age‐specific incidence of dementia for women and men. A probable reason for increased dementia risk in women may be increased life expectancy and biological differences (e.g., sex‐specific hormones).^88^, ^89^ Furthermore, women in the MENA region often face barriers to healthcare facilities, including financial restrictions, caregiving responsibilities, and cultural expectations that discourage health‐seeking.^90^ Women also have not had equal educational and occupational opportunities^91^ that might have negatively impacted cognitive reserve which contributes to the risk of dementia.^92^

We also have identified a slight increase in dementia prevalence with age across MENA countries, which is in agreement with some previous studies.^93^, ^94^ The prevalence of dementia is affected by the lifespan of individuals and the incidence rate of disease changes over time. The increased number of patients living with dementia in the MENA region might be partially because of changes in the age distribution of populations.^95^ Since increasing age is the greatest risk factor for dementia, the growing prevalence of disease in this region could be owing to the increased proportion of older individuals in the population.^76^ Moreover, a decline in fertility rates, improvements in healthcare systems, and consequently better prevention, diagnosis, and treatment of diseases have contributed to increasing life expectancy and accelerating population aging.^96^ However, longer life expectancy is accompanied by an increase in chronic diseases such as cardiovascular disease, diabetes, and neurodegenerative conditions such as AD.^97^Prior studies have proposed that there might be significant geographical differences in the prevalence and incidence of dementia. The strengths of this meta‐analysis included a large number of population‐based studies and the use of comprehensive analyses (e.g., subgroup and meta‐regression analyses). However, there are major limitations in the available data, such as a lack of nationally representative studies, few reports from some countries of the MENA (e.g., Iraq, Kuwait, Oman), and the marked heterogeneity between countries. Also, differences in screening tools, and inclusion and diagnostic criteria of the studies included in the meta‐analysis could influence the prevalence estimate. A major limitation of this study is the time‐varying prevalence rate and prolonged time span over which the included papers were published, with some dating back more than three decades. Given the scarcity of data on dementia epidemiology in the MENA region, which has been significantly understudied over the past few decades, excluding older publications could have introduced additional bias into the reported prevalence estimates. Finally, some factors associated with the prevalence of dementia, such as education level, gender, economic status, marital status, risk factors, and dementia subtypes were not assessed due to insufficient data.

## 5 CONCLUSION

In summary, this meta‐analysis showed that dementia is a common neurological condition among older adults living in the MENA region. However, slight differences in the prevalence of dementia between subgroups stratified by countries and sex were observed. The increasing number of aging populations and risk of dementia in this region underscores the need for developing novel therapies, as well as preventive measures, by targeting modifiable and region‐specific risk factors. Hence, currently available data are insufficient and more research on MENA countries is urgently needed to record and monitor trends in dementia prevalence and incidence, and to identify region‐specific dementia risk factors.

## CONFLICT OF INTEREST STATEMENT

All authors declare no financial or non‐financial competing interests related to this publication. Any other conflicts of interest are disclosed below.

S.M. is a full‐time employee of the Alzheimer's Association. The views and opinions expressed by authors in this publication represent those of the authors and do not necessarily reflect those of the Alzheimer's Association. AAH has received funding for research from the Medical Research Council, UK (grant MR/T005580/1) through the Clinical Academic Partnership Award, and the National Institute of Health/NIA, USA (grant 1R56AG074467‐01) through her institute. A.A.H. has received funding for Alzheimer's education, training and advice from Biogen, Eisai and Lilly. H.R.S. and R.N.M. report being a Director of SMarT Minds WA, Australia and have had or are receiving research support from Alzheimer's Research Australia as well as Pharmaceutical and Nutraceutical companies including Alector, Alnylam Pharmaceuticals, CWEKPTYLTD, WA, Australia, and Biogen pharmaceuticals.Author disclosures are available in the Supporting Information.

## Supporting information



Supporting information

Supporting information
