# Supplementary material for: Prevalence of dementia in selected Middle East and North Africa (MENA) countries: A systematic review and meta‐analysis
Source: Alzheimers Dement. 2026 Jan 28;22(1):e71109. doi: 10.1002/alz.71109 (PMC12851897; doi:10.1002/alz.71109)
Supplement: Supplementary file 2 — Supporting information [file ALZ-22-e71109-s001.docx]

| **Database** | **Keywords** |
| --- | --- |
| PubMed | (Middle East and North Africa[tw] OR Algeria[ad] OR Algeria[tw] OR Bahrain[ad] OR Bahrain[tw] OR Djibouti[ad] OR Djibouti[tw] OR Egypt[ad] OR Egypt[tw] OR Iraq[ad] OR Iraq[tw] OR Jordan[ad] OR Jordan[tw] OR Kuwait[ad] OR Kuwait[tw] OR Lebanon[ad] OR Lebanon[tw] OR Libya[ad] OR Libya[tw] OR Mauritania[ad] OR Mauritania[tw] OR Morocco[ad] OR Morocco[tw] OR Oman[ad] OR Oman[tw] OR Palestine[ad] OR Palestine[tw] OR Qatar[ad] OR Qatar[tw] OR Saudi Arabia[ad] OR Saudi Arabia[tw] OR Somalia[ad] OR Somalia[tw] OR Sudan[ad] OR Sudan[tw] OR Syria[ad] OR Syria[tw] OR Tunisia[ad] OR Tunisia[tw] OR United Arab Emirates[ad] OR United Arab Emirates[tw] OR Emirates[ad] OR Emirates[tw] OR Yemen[ad] OR Yemen[tw] OR Iran[ad] OR Iran[tw] OR Israel[ad] OR Israel[tw] OR Turkey[ad] OR Turkey[tw]) AND (Prevalen*[tw] OR inciden*[tw] OR epidemiolog*[tw]) AND (Alzheime*[tw] OR alzheimer[MH] OR dementia[MH] OR dement*[tw] OR "vascular dementia"[tw] OR "frontotemporal dementia"[tw] OR "dementia with Lewy bodies"[tw]) NOT (review[pt]) NOT ("rats"[tiab] OR "mice"[tiab] OR "animal"[tiab]) |
| Scopus | ( TITLE-ABS-KEY ( middle AND east AND north AND africa ) OR TITLE-ABS-KEY ( algeria ) OR TITLE-ABS-KEY ( bahrain ) OR TITLE-ABS-KEY ( djibouti ) OR TITLE-ABS-KEY ( egypt ) OR TITLE-ABS-KEY ( irag ) OR TITLE-ABS-KEY ( jordan ) OR TITLE-ABS-KEY ( kuwait ) OR TITLE-ABS-KEY ( lebanon ) OR TITLE-ABS-KEY ( libya ) OR TITLE-ABS-KEY ( mauritania ) OR TITLE-ABS-KEY ( morocco ) OR TITLE-ABS-KEY ( oman ) OR TITLE-ABS-KEY ( palestine ) OR TITLE-ABS-KEY ( qatar ) OR TITLE-ABS-KEY ( saudi AND arabia ) OR TITLE-ABS-KEY ( somalia AND plate ) OR TITLE-ABS-KEY ( sudan ) OR TITLE-ABS-KEY ( syria ) OR TITLE-ABS-KEY ( tunisia ) OR TITLE-ABS-KEY ( united AND arab AND emirates ) OR TITLE-ABS-KEY ( yemen ) OR TITLE-ABS-KEY ( iran ) OR TITLE-ABS-KEY ( turkey ) OR TITLE-ABS-KEY ( israel ) AND TITLE-ABS-KEY ( prevalen* ) OR TITLE-ABS-KEY ( inciden* ) OR TITLE-ABS-KEY ( epidemiolog* ) AND TITLE-ABS-KEY ( alzheime* ) OR TITLE-ABS-KEY ( dement* ) OR TITLE-ABS-KEY ( vascular AND dementia ) OR TITLE-ABS-KEY ( frontotemporal AND dementia ) OR TITLE-ABS-KEY ( dementia AND with AND lewy AND bodies ) ) AND ( LIMIT-TO ( DOCTYPE , "ar" ) OR LIMIT-TO ( DOCTYPE , "cp" ) OR LIMIT-TO ( DOCTYPE , "le" ) ) AND ( LIMIT-TO ( LANGUAGE , "English" ) ) AND ( LIMIT-TO ( SRCTYPE , "j" ) OR LIMIT-TO ( SRCTYPE , "p" ) ) |
| Web of science | TS= ("Middle East and North Africa" OR Algeria OR Bahrain OR Djibouti OR Egypt OR Iraq OR Jordan OR Kuwait OR Lebanon OR Libya OR Mauritania OR Morocco OR Oman OR Palestine OR Qatar OR "Saudi Arabia" OR Somalia OR Sudan OR Syria OR Tunisia OR "United Arab Emirates" OR Yemen OR Iran OR Turkey OR Israel) AND TS=(prevalence OR incidence OR epidemiology) AND TS=(Alzheimer OR dementia) |
